# ZC3H12A Expression in Different Stages of Colorectal Cancer

**DOI:** 10.18632/oncoscience.480

**Published:** 2019-04-02

**Authors:** Tao Chen, Di Du, Jian Chen, Pinghong Zhou, John N. Weinstein, Liqing Yao, Yuexin Liu

**Affiliations:** ^1^ Endoscopy Center and Endoscopy Research Institute, Zhongshan Hospital of Fudan University, Shanghai, China; ^2^ Department of Bioinformatics and Computational Biology, The University of Texas MD Anderson Cancer Center, Houston, Texas, USA; ^3^ Department of Gastroenterology, Hepatology & Nutrition, The University of Texas MD Anderson Cancer Center, Houston, Texas, USA

**Keywords:** ZC3H12A, Colorectal Cancer, biomarker, early detection

## Abstract

Identification of CRC patients with early-stage disease provides the opportunity for curative local resection. However, robust markers for stage I tumor prediction are yet to be developed. We analyzed RNA-sequencing data of 221 CRC samples using the TCGA dataset to identify novel biomarkers for stage I CRC. We next validated the TCGA finding in an independent GEO cohort of 290 CRC patients and in a third cohort of 110 CRC tumors and matched normal samples. We further performed correlative analysis of ZC3H12A gene expression with clinicopathologic features and disease-free survival. Expression correlation of ZC3H12A with the chemokine ligands was evaluated via Student’s t-test. In the TCGA cohort, stage I CRC patients had significantly higher ZC3H12A mRNA expression as compared with the other three stages combined and with the other individual stages in a pairwise manner (P<0.001 for all comparisons). The significant association of ZC3H12A gene expression with stages was further validated in the GEO cohort and in the additional third cohort. In support of these findings, we further found that patients with lower ZC3H12A expression had more aggressive tumor features and shorter disease-free survival. Biologically, ZC3H12A expression was significantly correlated with expression of three chemokine ligands (CXCL1, CXCL2 and CXCL3), suggesting that immune response dysregulation likely contributes to CRC development. Our results demonstrate ZC3H12A’s potential role in identification of CRC patients with early-stage disease.

## INTRODUCTION

Colorectal cancer (CRC) is the third most common malignancy after breast and lung cancer and contributes to the third cancer-related death. In the United States, more than 135,000 new cases and more than 50,000 deaths were estimated in 2017 [1]. Early-detection strategies are in dire need of improvement because the lethality of CRC patients is primarily due to the advanced-stage diseases at the time of diagnosis. Due to lymph-node or distant metastasis, these patients have limited opportunities for curative local resection, including endoscopic mucosal resection, a novel minimally invasive methodology for early CRC. Different from Stage I patients who are treated by endoscopic muscosal resection, patients with late- stage disease are typically treated with radical surgery, postoperative radiotherapy and/or chemotherapy with strong cytotoxicity but modest benefits [2, 3]. The current standard of care for determination of TNM stage is by surgery. Therefore, early stratification of colorectal cancer from tissue biopsies will help oncologists more accurately select patients who should have extensive surgical staging procedures or receive systemic adjuvant therapy, and thus represents a key to reducing the morbidity and mortality associated with CRC.

Colonoscopy screening is typically used for the early detection of CRC and contributes to a decrease in CRC-related mortality, however, its reach is limited by poor patient compliance [4, 5]. A variety of research has been directed in the past to search for prognostic and predictive biomarkers in CRC [6-13], but with relatively weak discriminatory power and limited generalizability [8, 10]. Optimized marker panels are yet to be developed. The lack of reproducibility may be attributable to the sizes of the analyzed cohorts, a limited number of candidate mRNAs, or the methods of assessing marker quantities.

In this study, we sought to overcome those limitations in identifying a marker of early CRC. We utilized a large population of samples, over 600 CRC cases, to identify and validate a gene expression biomarker, ZC3H12A (zinc finger CCCH-type containing 12A), for prediction of stage I CRC. It was previously reported that ZC3H12A expression was correlated with tumor grade and patient survival in clear cell renal cell carcinoma [14] and breast cancer [15]. ZC3H12A suppressed tumor progression or metastasis either by inducing apoptosis [15] or by inhibiting angiogenesis or the epithelial-to-mesenchymal transition (EMT) signaling axis [14, 16]. In the current study, four different methods were used to measure the gene expression quantities. RNA-sequencing data from The Cancer Genome Atlas (TCGA) were used in the discovery phase, and microarray data from a publicly available database were used for validation. Single-gene expression measurements such as real-time PCR and immunohistochemical assays were used in a third CRC cohort to evaluate clinical utility of the identified candidate biomarker. The biological functions of the identified marker gene are also interpreted. Our aim is to investigate ZC3H12A’s potential role in identification of CRC patients with early-stage disease.

## RESULTS

### Identification of *ZC3H12A* as a candidate biomarker for early-stage CRC in the discovery set

**Figure 1:**
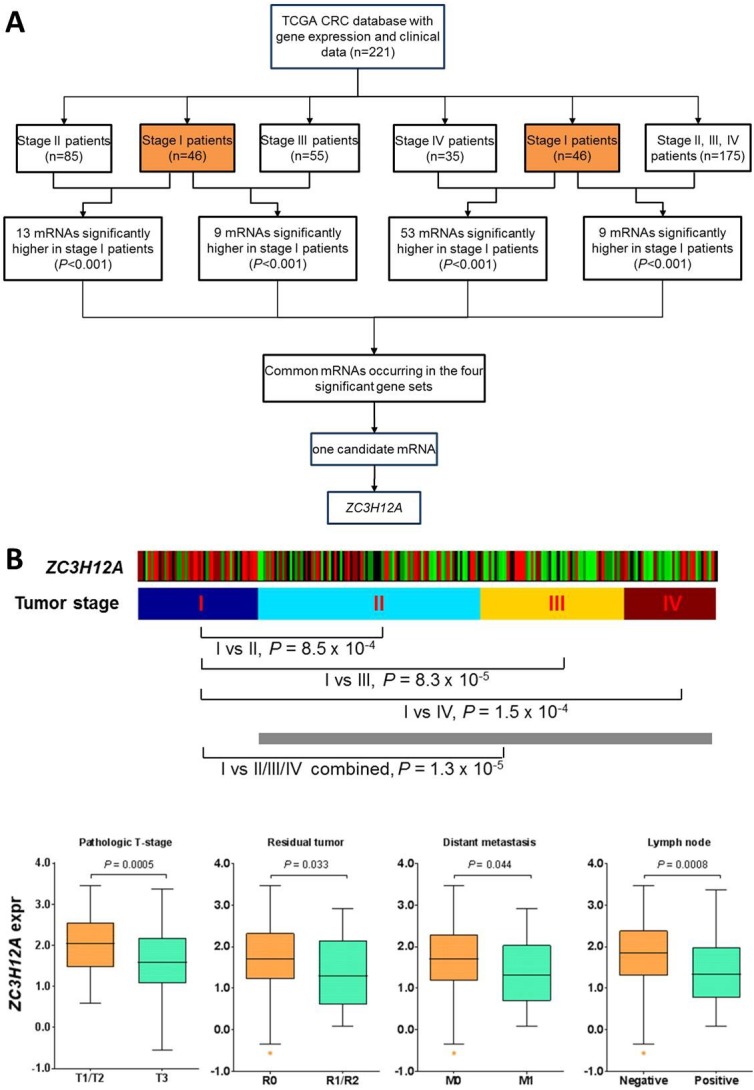
**(A)** Selection of CRC early-stage markers in the discovery set from The Cancer Genome Atlas. **(B)** Correlation of *ZC3H12A* mRNA expression with CRC tumor stage and other clinical features in the TCGA dataset. Patients were first dichotomized into two groups based on other clinical features, in particular for primary tumor pathologic spread (T stage), T1/T2 vs T3; for residual tumor, R0 vs R1/R2; for distant metastasis, M0 vs M1; and for lymph node, negative vs positive. Statistical difference in *ZC3H12A* expression between the dichotomic groups is assessed via Student’s t-test and indicated in the box-and-whiskers plots (center line, median; box limits, upper and lower quartiles; whiskers 1.5 x interquartile range; and points, outliers). Missing values were excluded from statistical test.

RNA-sequencing data and clinical annotations of total 221 CRC samples were obtained from the TCGA data portal. In this discovery set, the median age is about 70 years (range, 35 to 90). 20.8% were stage I tumors and the other 79.2% were either stage II, III or IV tumors, specifically 46 stage I, 85 stage II, 55 stage III and 35 stage IV cases (Table 1). To identify a molecular biomarker specific for stage I tumors, we performed several statistical comparisons. In particular, we compared stage I tumors versus stage II, III, and IV patients, and identified 13, 9, and 53 mRNAs, respectively, that were significantly higher in stage I patients (Figure 1A, P < 0.001, Student’s t test). Then we combined stage II, III, and IV patients and identified 9 mRNAs that were significantly higher in stage I patients as compared to this combined group (Figure 1A, P<0.001). Detailed information of these significantly higher gene lists was provided in (Supplementary Table 1). Next we carried out overlapping analysis to find common mRNAs that occurred in these four significantly higher gene sets. We found only one candidate mRNA: ZC3H12A. Consistent with its tumor stage association, patients with lower ZC3H12A expression also had more aggressive tumor features in the TCGA CRC cohort, including tumor pathologic T stage (P = 0.0005), residual tumor (P = 0.033), distant metastasis (P = 0.044) and positive lymph nodes (P = 0.0008) (Figure 1B). Because the median follow-up duration was very short in the TCGA cohort [17], we did not perform correlative analysis of ZC3H12A expression with survival. No significant correlation was observed for patient age (P = 0.4565, Student’s t test), gender (P = 0.0798), tumor site (P = 0.5701) and MSI status (P = 0.3958) (Table 2).

**Table 1:** Clinicopathologic characteristics of colorectal cancer patients in the training (TCGA), validation (GEO), and Shanghai cohorts.*

	TCGA (n = 221)	GSE14333 (n = 290)	Shanghai (n = 110)
**Age, years**			
Mean [SD] Median [Range]	69.4 [11.6] 70 [35 – 90]	66.0 [12.5] 67 [26 – 92]	64.0 [11.9] 66 [31 – 85]
**Gender**			
Female Male	106 (48.0) 115 (52.0)	126 (43.4) 164 (56.6)	44 (40.0) 66 (60.0)
**Stage§**			
I (A) II (B) III (C) IV (D)	46 (20.8) 85 (38.5) 55 (24.9) 35 (15.8)	44 (15.2) 94 (32.4) 91 (31.4) 61 (21.0)	7 (6.4) 52 (47.3) 45 (40.9) 6 (5.4)
**Tumor site**			
Colon Rectum Unknown	153 (69.2) 68 (30.8) 0	250 (86.5) 39 (13.5) 1	110 (100) 0 0
**MSI Status**			
MSI‡ MSS Unknown	65 (29.5) 155 (70.5) 1	NA NA NA	NA NA NA

**TCGA, The Cancer Genome Atlas; SD, standard deviation; MSI, microsatellite instability; MSS, microsatellite stable; NA, not applicable.**

* Values are reported as No. (%). Missing values are excluded from percentage calculation and statistical test.

§ In the GSE14333 cohort, tumors were staged based on Dukes staging system.

‡ Including both MSI-H and MSI-L cases.

**Table 2:** Clinical significance of ZC3H12A in colorectal cancer specimens from the TCGA, GSE14333, and Shanghai databases.

Platform	TCGA Cohort		GSE14333		Shanghai Cohort	
	RNA-sequencing		Microarray		rt-PCR	
Variables	Mean ± SDa	Pb	Mean ± SDa	Pb	Mean ± SDa	Pb
**Age, years**						
<= 70	1.654 ± 0.780	0.456	7.489 ± 0.831	0.325	−7.351 ± 0.084	0.500
> 70	1.737 ± 0.866		7.589 ± 0.863		−7.254 ± 0.117	
**Gender**						
Female	1.593 ± 0.852	0.080	7.479 ± 0.820	0.372	−7.406 ± 0.115	0.288
Male	1.787 ± 0.786		7.569 ± 0.863		−7.257 ± 0.084	
**Tumor site**						
Colon	1.673 ± 0.825	0.570	7.529 ± 0.847	0.809	−7.317 ± 0.068	−
Rectum	1.741 ± 0.820		7.564 ± 0.826			
**MSI status**						
MSIc	1.622 ± 0.083	0.396	−	−	−	
MSS	1.726 ± 0.812		−		−	

**TCGA, The Cancer Genome Atlas; SD, standard deviation; MSI, microsatellite instability; MSS, microsatellite stable.**

^a^ Data were presented as mean and standard deviation of *ZC3H12A* mRNA expression in each of the specified groups.

^b^ Student’s t test.

^c^ Including both MSI-H and MSI-L cases.

### Surrogate validation of *ZC3H12A* in a second large cohort of CRC patients

**Figure 2:**
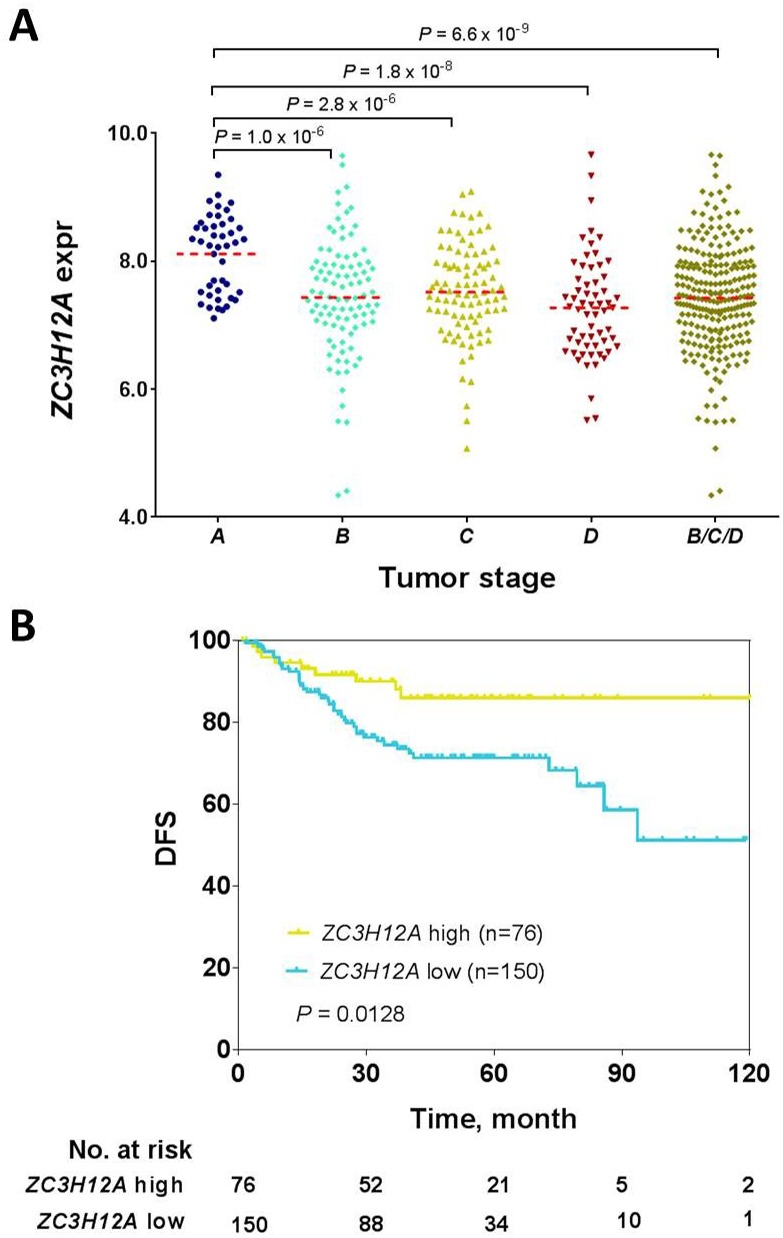
Correlation of ZC3H12A mRNA expression with CRC tumor stage **(A)** and patient disease-free survival **(B)** in the validation cohort (GSE14333). Statistical significance in ZC3H12A mRNA expression was assessed via Student’s t-test. The number of patients at various time points are shown below each curve. Note that patients in this cohort were staged based on Duke staging system and 64 patients had no survival data or vital status in this cohort.

To validate the results as presented, we obtained an external dataset comprising 290 CRC cases from GEO (GSE14333). Gene expression data in this validation cohort had been profiled via the Affymetrix microarray platform and tumors had been categorized as stage A, B, C and D following the Dukes staging system. Approximately 15% of the validation cohort was stage A, and patients in this cohort had a significantly younger median age than those in the TCGA cohort (Table 1, P = 0.0023, Mann-Whitney test). Correlation of *ZC3H12A* expression with tumor stage showed that *ZC3H12A* had significantly higher expression in stage A tumors than in stage B tumors (P = 1.0E-06), stage C tumors (P = 2.8E- 06), stage D tumors (P = 1.8E-08) and stage B, C and D tumors combined (P = 6.6E-09) (Figure 2A), confirming that *ZC3H12A* is a robust biomarker for early-stage CRC detection regardless of gene expression measurement method. Also consistent with the results discovered from the TCGA cohort, *ZC3H12A* expression was not correlated with patient age (P = 0.3249, Student’s t test), gender (P = 0.3722) or tumor site (P = 0.8093) in the validation cohort (Table 2). Although other clinicopathological features such as T stage, lymph node status and distant metastasis were not available for patients in this validation cohort, we found that *ZC3H12A* expression was associated with significantly longer disease-free survival (DFS) with a hazard ratio for relapse of 0.41 (95% confidence interval 0.27- 0.85; P = 0.0128, log-rank test) (Figure 2B), which is consistent with the observed association of lower *ZC3H12A* expression with more aggressive tumor features in the TCGA cohort. Of note, the median DFS was not reached in both groups. We repeated this survival analysis, separately, within the stage A, or B, or C patients (Supplementary Figure 1). For the stage A or B patients who typically had favorable prognosis, those with higher *ZC3H12A* expression exhibited a trend toward a better survival as compared to those with lower *ZC3H12A* expression, though the statistical significance was not reached likely because of smaller size of analyzed samples used in this analysis. Furthermore, multivariate Cox proportional hazards model analyses showed that ZC3H12A expression was significantly correlated with survival independent of patient sex and age (Supplementary Table 2), but not of tumor stage (Supplementary Table 3). Taken together, these data collectively suggested that *ZC3H12A* is a potent and independent biomarker for early-stage CRC.

### ZC3H12A is involved in the immune response process, contributing to CRC development

**Figure 3:**
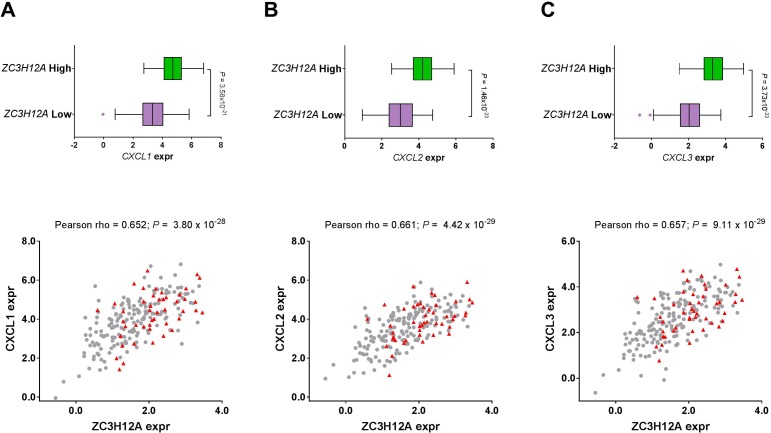
Correlation of *ZC3H12A* mRNA expression with the expression levels of three chemokine ligands in the TCGA CRC patient cohort: **(A)** *CXCL1*, **(B)** *CXCL2* and **(C)** *CXCL3*. The top panel shows the expression difference of those chemokine ligands between *ZC3H12A* high group and *ZC3H12A* low group while patients were categorized into these two groups based on the median *ZC3H12A* expression. In the bar-and-whiskers plots, the box-plot elements are defined as: center line, median; box limits, upper and lower quartiles; whiskers 1.5 x interquartile range; and points, outliers. The bottom panel shows the expression correlation of *ZC3H12A* with the chemokine ligands while patients with stage I disease were indicated in red and those with stage II, III, or IV were indicated in gray.

To characterize the biological properties of the *ZC3H12A* gene, we first calculated the expression correlation of *ZC3H12A* with all other genes in the genome, and then ranked the genes in descending order based on the correlation coefficients. Gene set enrichment analysis (GSEA) on the genome-wide correlation profile showed that the top-ranked gene sets were, to a certain extent, related to immune response, suggesting that *ZC3H12A* is likely involved in immune or inflammatory response. Close examination of the ranked gene list found that the three chemokine ligands (*CXCL1*, *CXCL2* and *CXCL3*) were among the top 10 most significantly correlated genes (Supplementary Table 4). Moreover, these ligands are members of a gene family, and therefore assume to share common protein structural domains and demonstrate functional redundancy. The functional role of these ligands in immune or inflammatory response is well consistent with the GSEA result. As a consequence, we next investigated these three ligands more in details. Expression levels of three chemokine ligands (*CXCL1*, *CXCL2* and *CXCL3*) were strongly and significantly correlated with *ZC3H12A* expression. Moreover, these three ligands were significantly higher in the group with high *ZC3H12A* expression compared with the group with low *ZC3H12A* expression (Figure 3). These data suggest that *ZC3H12A* is involved in the immune or inflammatory response process, which likely contributes to CRC development. Also in support of this observation is the significant and positive correlation of *ZC3H12A* with the immune checkpoint molecules, PD-1 and PD-L1 (Supplementary Table 5). Different from breast or renal cancer, ZC3H12A less likely induces apoptosis [15] or inhibits angiogenesis [14] in CRC indicated by expression correlation of ZC3H12A mRNA with those processes- related genes (Supplementary Tables 6 & 7).

### Clinical implications of ZC3H12A as a biomarker for early-stage CRC

**Figure 4:**
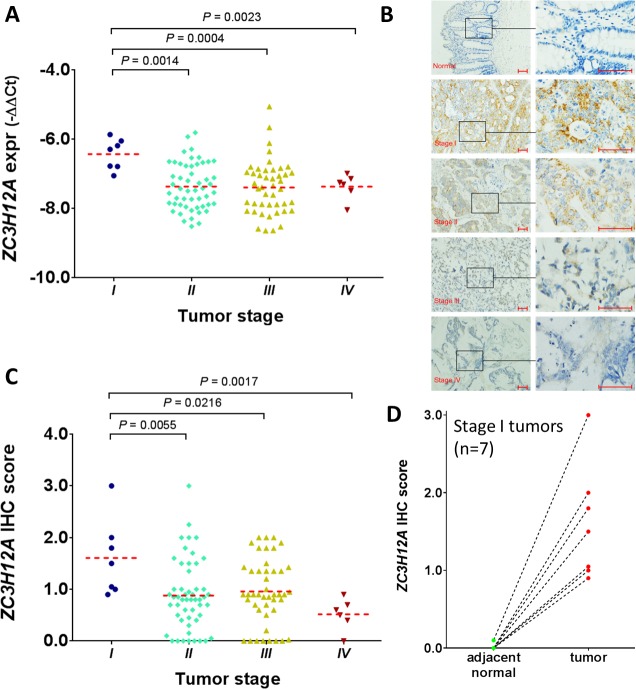
Examination of ZC3H12A expression in the CRC patient cohort at Zhongshan Hospital, Shanghai **(A)** Correlation of *ZC3H12* expression with tumor stage at the transcriptomic level. The *ZC3H12* mRNA expression was measured via quantitative real-time PCR assay. The data were log-transformed before p-value calculation. Each dot represents average value of three biological replicates. **(B)** Representative IHC images of ZC3H12A-stained adjacent normal tissue and tumor tissues at different stage (magnification: x100 and x400). Scale bars: 50 µm. **(C)** Correlation of ZC3H12 expression with tumor stage at the proteomic level. The ZC3H12 protein expression was measured via immunohistochemical assay. **(D)** ZC3H12A protein expression between stage I tumors and adjacent normal tissue. The dotted lines indicate samples from the same patient.

Although the gene expression data were measured by RNA-sequencing and a microarray platform in the training and validation cohorts, these high-throughput techniques are not applicable in clinical practice for single- gene measurement. To determine the clinical utility of the candidate biomarker, we next performed real-time PCR assays for the *ZC3H12A* gene and actin on 110 CRC cases obtained at Zhongshan Hospital in Shanghai, China. In our cohort, all patients had colon cancer and approximately 6.4% had stage I disease. In addition, the patient ages were significantly younger in our cohort than in the TCGA cohort (P = 0.0002, Mann-Whitney test), but no significant difference in patient age was observed between our cohort and the GEO cohort (P = 0.1645) (Table 1). The *ZC3H12A* mRNA expression was significantly higher in stage I tumors than in stage II tumors (P = 0.0014), stage III tumors (P = 0.0004) and stage IV tumors (P = 0.0023), further confirming its predictive role as a biomarker of early-stage CRC tumors (Figure 4A). Next, we performed IHC assays on the same tumor specimens and adjacent normal tissues (Figure 4B). For the purpose of clarity, an enlarged version of this panel is provided in the Supplementary Figure 2. Indeed consistent with its transcriptome level, ZC3H12A protein was significantly higher in the stage I tumors than in stage II tumors (P = 0.0055), stage III tumors (P = 0.0216) and stage IV tumors (P = 0.0017) (Figure 4C). In addition, we calculated the sensitivity, specificity, positive-predictive value and negative-predictive value by using ZC3H12A expression to predict CRC patients with stage I disease in the TCGA, GSE14333, and Shanghai cohorts (Supplementary Table 8). Regardless of the different data measurement platforms, ZC3H12A demonstrated the potential usefulness as a biomarker for early diagnosis of patients with colorectal cancer. Notably, among the 7 matched stage I tumor and normal tissue samples, none had detectable expression of ZC3H12A protein in normal tissue, while all 7 patients had significantly higher expression of ZC3H12A protein in the stage I tumors as compared to matched normal samples (P = 0.0156, Wilcoxon matched-pairs signed rank test) (Figure 4D). In support of this observation, we obtained the gene expression data from the TCGA PanCanAtlas freezed data sets [18, 19] including 595 CRC tumors (103 stage I, 228 stage II, 176 stage III, and 88 stage IV) and 51 adjacent normal tissues. Consistent with the ZC3H12A protein data, stage I tumors had significantly higher *ZC3H12A* mRNA expression than adjacent normal tissues (Supplementary Figure 3). Even with this much larger CRC patient cohort, *ZC3H12A* expression remains significantly higher in stage I tumors as compared to stage II, or III, or IV tumors, further confirming findings from the original TCGA cohort (Supplementary Figure 3).

## DISCUSSION

CRC remains a disease with high incidence and mortality throughout the world, despite continuous advancement in diagnostic and therapeutic methods [7]. Because of the strong dependence of prognosis on stage at diagnosis, early detection of CRC has great potential to improve the rate of curative resection and reduce the burden of this disease. In this study, we have, for the first time, to our knowledge, identified and validated *ZC3H12A* as a promising and robust biomarker for CRC stage I patients. Biologically, strong correlation of ZC3H12A with genes pertaining to immune response suggests that ZC3H12A plays a functional role in cellular inflammation, which is likely attributable to CRC pathogenesis. Results from single-gene measurements demonstrated the *ZC3H12A* gene’s translational potential as a marker for early-stage CRC in the clinical setting.

The *ZC3H12A* gene is a key molecule in the regulation of immune response [20], and targeted disruption of Zc3h12a results in fatal inflammatory disease [21]. Endoribonuclease ZC3H12A (also known as monocyte chemoattractant protein-induced protein 1, MCPIP1 and Regnase-1) encoded by *ZC3H12A*, possesses ribonucleolytic activity and destabilizes mRNAs of inflammatory modulators such as IL-1β [22], IL-6 [23], IL-12b [24], IL-2 [25], c-Rel [25] and Ox40 [25] transcripts, through cleavage of their 3’ untranslated regions. Consistent with the current study, a gene expression signature identified from peripheral blood mononuclear cells to discriminate patients with CRC from subjects without the lesions was significantly enriched in biological functions related to inflammatory and immune response.[10] On the other hand, *ZC3H12A* gene could be activated by pro-inflammatory stimuli, including IL- 1β, IL-17, TNF-α, phorbol myristate acetate [22, 26, 27] and lipopolysaccharides in macrophages and hepatocytes [22, 28].

ZC3H12A has been shown to induce apoptosis in breast cancer cells by degrading the mRNA of anti- apoptotic genes through binding to a stem-loop structure in the 3’UTR of target transcripts [15]. Our results show that it is less likely the case in colorectal cancer cells. ZC3H12A neither suppresses the expression (in terms of negative expression correlation) of those previously reported anti-apoptotic gene transcripts [15] (including *BCL2L1*, *BCL2A1*, *RELB*, *BIRC3* and *BCL3*) nor enhances the expression (in terms of positive expression correlation) of those pro-apoptotic gene transcripts [15] (including *BAD*, *RIPK2*, *FAS* and *DEDD2*) (Supplementary Table 6). It has also been demonstrated that ZC3H12A promotes angiogenesis by increasing the expression of genes encoding vascular endothelial growth factor (*VEGFA*) in human umbilical vein endothelial cells [29, 30]. Consistent with these prior works, *ZC3H12A* expression is significantly and positively correlated with *VEGFA* expression in CRC (Supplementary Table 7). The discrepancy is primarily due to tumor site specificity. Likely for the same reason, our results show that *ZC3H12A* is not the negative regulation of the proinflammatory cytokines (i.e., *IL6*, *IL8*) in CRC (Supplementary Table 7), different from what was reported in renal cell carcinoma. [14] Instead, our results demonstrate a strong and positive correlation of *ZC3H12A* gene with the chemokine ligands (i.e., *CXCL1*, *CXCL2*, and *CXCL3*). These chemokine ligands function as attracting leukocytes to inflammatory sites and contribute significantly to tumor initiation and development. In addition, these ligands may lead to tumor regression in CRC patients by activating immune response through chemoattraction of leukocytes [31]. The functional role of these ligands in immune response activation along with their positive correlation with ZC3H12A gene, indicates that CRC patients with lower ZC3H12A expression evade immune surveillance, therefore exhibiting worse prognosis and more aggressive tumor features as demonstrated in this study. However, the causal relationship between ZC3H12A and these ligands remains elusive and certainly deserves in-depth investigation in the follow-up study.

Our results also showed that patients with lower *ZC3H12A* gene expression had more aggressive tumor features and that lower *ZC3H12A* expression was associated with significantly shorter disease-free survival. *ZC3H12A* is located on chromosome 1 (1p34.3), which is frequently deleted in CRC [17]. Consistent with our study, the TCGA Research Network found that deletion of this region was significantly associated with advanced tumor stage, lymph-node invasion, vascular invasion and tumor metastasis, although the underlying mechanism remains elusive. The coordinate correlation of *ZC3H12A* expression levels with tumor stage and prognosis presented in this study provides a potential mechanism underlying that chromosomal association.

Our study is not without limitations. The association of ZC3H12A expression with CRC tumor stage should be validated in large prospective trials before it can be translated into clinical practice. Furthermore, functional studies are required in order to fully understand and exploit the role of ZC3H12A for immune response activation in early-stage CRC. In addition, we used paired noncancerous adjacent matched tissues of patients with CRC as the control.

In summary, ZC3H12A is elevated in stage I CRC patients and correlated with tumor prognosis likely due to its involvement in immune or inflammatory process. The findings may have potential clinical implications in patient management and shed new light on CRC tumor biology.

## MATERIALS AND METHODS

### Patient samples

The training dataset, consisting of 221 CRC samples with clinical annotation and gene expression data, was obtained from TCGA [17] and is available at the TCGA data portal (https://gdc.cancer.gov). Tumors in this cohort were staged based on the tumor-node-metastasis (TNM) staging system. The microsatellite instability (MSI) status was determined by evaluating a panel of seven markers. Tumors with no altered markers were classified as microsatellite-stable (MSS), tumors with one to four altered markers as low levels of MSI (MSI-L), and tumors with five to seven altered markers as high levels of MSI (MSI-H). In addition, tumors were classified as R0, R1 and R2 based on the residual tumor sites [17]. The access to the TCGA database was approved by the National Cancer Institute.

We further obtained a validation cohort comprising a total of 290 CRC cases from Gene Expression Omnibus (GEO), accession no. GSE14333 [32]. The median age of this cohort is 67 years (range, 26 to 92). Tumors in this cohort were staged based on Dukes staging system; 15.2% were stage A tumors and the rest (84.8%) were stage B, C, or D tumors. Disease-free survival (DFS) was defined as the interval from surgery to the first relapse. Of note, this is a surrogate validation because the staging systems are different in the TCGA and GEO cohorts.

An additional 110 tumor samples and paired noncancerous adjacent matched tissues were obtained from patients with CRC treated at Zhongshan Hospital, Shanghai, China, between 2008 and 2011. All experiments were performed in accordance with relevant guidelines and regulations. Patients with radiotherapy or chemotherapy treatment before surgery were excluded. TNM staging was performed according to American Joint Committee on Cancer standards. All patients in this cohort had colon cancer. The majority of tumors in this cohort were stage II or III tumors and approximately 6.4% were stage I.

The study was approved by the institutional review board at the University of Texas MD Anderson Cancer Center and at Zhongshan Hospital, Fudan University. Written consent was obtained from all live patients.

### Gene expression analysis and gene set enrichment analysis

Gene expression data from TCGA were generated using the RNAseq platform (Illumina GAII sequencers). Data, represented as RPKM (reads per kilobase per million), were first median centered across the cohort and then log transformed [17]. Genes with smaller RPKM value (<0.5) in at least one-fourth of the samples were first excluded. To identify a molecular biomarker specific for stage I tumors, we performed several statistical comparisons via the significant analysis of microarrays method: stage I versus stage II/III/IV combined and stage I versus stage II or III or IV in a paired manner. The mean expression difference and test statistic were provided for all of the genes and for all of these analyses. Genes with inconsistent expression difference among these analyses were excluded. Next, we applied the statistical cutoff of P < 0.001 to all analyses, and the gene that satisfied all these criteria was considered a stage I-specific biomarker candidate.

The molecular biomarker was further validated in an independent dataset (GSE14333). The gene expression data in the validation set were profiled using Affymetrix Human Genome U133 Plus 2.0 arrays and preprocessed as previously described [32]. For genes associated with multiple probes, an average was taken as the gene expression value.

To characterize the biological properties of the *ZC3H12A* gene, we first calculated the expression correlation of the *ZC3H12A* gene with each of the other genes in the genome and then ranked the genes in descending order based on the correlation scores. Then we used gene set enrichment analysis (GSEA) to associate the genes with the gene ontology. Gene ontology terms with overrepresentation of the genes that were positively correlated with *ZC3H12A* were then determined [33].

### Quantitative real-time PCR assay

To evaluate clinical utility of the identified candidate biomarker, we performed the quantitative real-time polymerase chain reaction (PCR) assay on a third patient cohort of 110 CRC cases obtained from Zhongshan Hospital, Shanghai. Total RNA was isolated using TRIzol reagent (Invitrogen) according to the manufacturer’s instructions. The amount of used total RNA was 20ng. TaqMan real-time PCR assays for ZC3H12A (forward, 5’-TGACGGGATCGTGGTTTCCAAC-3’, and reverse, 5’-GGCATCCACTTGTCATTGACGAAGG-3’) and actin (forward, 5’-GAAGAGCTACGAGCTGCCTGA-3’, and reverse, 5’-CAGACAGCACTGTGTTGGCG-3’) were from Takara Bio (SYBR® Premix Ex Taq™ II). The qPCR cycler, Roche Light Cycler 480II, was employed and the annealing temperature was 60 degree. All reactions, including the no-template controls, were run in triplicate. After the reactions were completed, the threshold cycle (Ct) values were determined using fixed threshold settings. The range of the obtained Ct values of ZC3H12A was 20.6-27.7. Data were analyzed using the 2^−ΔΔCT ^method [34] and log-transformed before statistical calculation.

### Immunohistochemical assay

Consecutive sections of 110 formalin-fixed, paraffin- embedded tumors from the same CRC patients as being used in the real-time PCR measurement were subjected to immunohistochemical (IHC) analysis for the ZC3H12A protein. Primary antibody, rabbit polyclonal ZC3H12A (Abcam; ab197976; 1:100 dilution), was used 30 minutes at room temperature. DAKO EnVision™+/HRP was used 30 minutes at room temperature, followed by DAB staining 5 minutes. The staining intensity of ZC3H12A was graded on a scale of 0 to 3 (0 for no staining, 1 for light yellow, and 3 for brown). The total score was calculated by the staining intensity multiplying the percentage of cells with immunoreactivity. The staining results were scored by two pathologists blinded to the clinical data.

### Statistical analysis

Statistical analysis was performed using Matlab 8.4 (MathWorks, Natick, MA), GraphPad Prism 5.0 (GraphPad Software Inc, La Jolla, CA) or SPSS 18.0 (SPSS Inc., Chicago, IL) as needed. Results were expressed as mean ± standard deviation. In the bar-and- whiskers plot, the box extends from the lower quartile to upper quartile the line in the middle is plotted at the median. The whiskers are 1.5 times interquartile range and individual points represent outliers. Student’s t-test was used to evaluate statistical differences in ZC3H12A expression between unpaired groups. Wilcoxon matched- pairs signed rank test was used to compare ZC3H12A expression between the stage I tumors and the adjacent normal samples. The median age difference among different datasets was assessed via Mann-Whitney U test. Pearson correlation analysis was used to correlate *ZC3H12A* mRNA expression with those of the three chemokine ligands (CXCL1, CXCL2 and CXCL3) with Student’s *t*-test. The Kaplan-Meier method was used with a log-rank test to assess survival difference between low- and high-ZC3H12A expression groups in the validation cohort. All experiments were performed in triplicate. All statistical tests were two-sided, and a P-value < 0.05 was considered statistically significant.

## SUPPLEMENTARY MATERIALS TABLES AND FIGURES


