# Supplementary material for: ZC3H12A Expression in Different Stages of Colorectal Cancer
Source: Oncoscience. 2019 Apr 2;6(3-4):301–11. doi: 10.18632/oncoscience.480 (PMC6508193; doi:10.18632/oncoscience.480)
Supplement: Supplementary file 1 [file oncoscience-06-301-s001.pdf]

SUPPLEMENTARY MATERIALS

Supplementary Table 1. Significantly higher gene expression list in Stage I patients as compared to the other groups of patients.

I vs II		I vs III		I vs IV		I vs II/III/IV combined	
Gene	P value	Gene	P value	Gene	P value	Gene	P value
ALG8	1.22E-04	TNFRSF10C	6.68E-05	CIITA	4.71E-05	ZC3H12A	1.30E-05
PPT2	1.97E-04	ZC3H12A	8.34E-05	LAP3	6.41E-05	ZNF599	0.0002452
ANKS1A	2.55E-04	ALPK1	3.33E-04	LOC165186	6.49E-05	BTBD6	0.0005632
ZNF689	2.64E-04	BTBD6	4.30E-04	GBP4	6.61E-05	SE1L3	0.0005938
ZNF599	3.50E-04	DDX5	4.66E-04	WARS	8.55E-05	SFRS11	0.0007216
TEAD3	3.90E-04	MGC16169	5.05E-04	CYB5D1	1.00E-04	IL17A	0.0007663
KIAA1505	4.64E-04	WRN	6.86E-04	GNLY	1.02E-04	TRBV3-1	0.0007829
CDYL	6.31E-04	C9orf128	8.96E-04	IQCG	1.13E-04	USP1	0.0008624
KIAA0467	6.38E-04	COL9A2	9.86E-04	INDO	1.23E-04	TTC21A	0.0008876
ZC3H12A	8.53E-04			FAM73A	1.31E-04		
RING1	9.46E-04			TTC21A	1.43E-04		
PALB2	9.47E-04			ZC3H12A	1.52E-04		
ADNP	9.88E-04			TRBV3-1	1.57E-04		
				RASGRP1	1.76E-04		
				C14orf161	2.36E-04		
				BIRC3	2.56E-04		
				CTLA4	3.11E-04		
				C9orf116	3.28E-04		
				GBP1	3.29E-04		
				EPHA10	3.93E-04		
				ICOS	4.14E-04		
				CPT1B	4.38E-04		
				ST3GAL5	4.77E-04		
				IL2RB	4.79E-04		
				SOCS1	5.00E-04		
				ENOSF1	5.23E-04		
				STAT1	5.37E-04		
				CD7	5.64E-04		
				IFNG	5.65E-04		
				CFB	5.69E-04		
				INDOL1	5.73E-04		
				STAT4	5.75E-04		
				PARP9	5.81E-04		
				FOXP3	6.31E-04		
				AGL	6.41E-04		
				KLRC1	6.46E-04		
				NLRC5	6.72E-04		
				GBP5	7.10E-04		
				TRPC7	7.18E-04		
				DDX5	7.36E-04		
				DAPP1	7.36E-04		
				UBASH3A	7.67E-04		
				FLJ25791	7.69E-04		
				TMEM173	7.82E-04		
				LAIR2	8.35E-04		
				SOCS6	8.39E-04		
				TYMS	8.49E-04		
				NFKBIZ	8.65E-04		
				WDR7	8.94E-04		
				TC2N	9.42E-04		
				LAG3	9.52E-04		
				JAK2	9.77E-04		
				CASP7	9.79E-04		

SUPPLEMENTARY MATERIALS

Supplementary Table 2. Multivariable models for disease-free survival in the GSE14333 sample cohort without tumor stage as covariate.

		HR(95%CI)	*P* value*
ZC3H12A expression§			
	High	1 [Ref]	
	Low	2.367 (1.145 – 4.893)	0.020
Sex			
	Female	1 [Ref]	
	male	1.110 (0.630 – 1.957)	0.718
Age increase of 1.0 year		0.984 (0.964 – 1.004)	0.123

HR: hazard ratio; CI: confidence interval.
* *P* values were derived from the Cox regression model including all variables in the table. The tests were two-sided.
§ ZC3H12A expression high or low group was the same as defined in Figure 2B.

Supplementary Table 3. Multivariable models for disease-free survival in the GSE14333 sample cohort including tumor stage as covariate.

		HR(95%CI)	*P* value*
ZC3H12A expression§			
	High	1 [Ref]	
	Low	1.751 (0.836 – 3.667)	0.137
Tumor Stageζ			
	A	1 [Ref]	
	B	2.834 (0.631 – 12.733)	0.174
	C	7.575 (1.773 – 32.366)	0.006
Sex			
	Female	1 [Ref]	
	male	1.019 (0.573 – 1.812)	0.948
Age increase of 1.0 year		0.990 (0.970 – 1.011)	0.363

HR: hazard ratio; CI: confidence interval.
* P values were derived from the Cox regression model including all variables in the table. The tests were two-sided.
§ ZC3H12A expression high or low group was the same as defined in Figure 2B.
ς Stage D was not included because the DFS data were not available.

SUPPLEMENTARY MATERIALS

Supplementary Table 4. The top 50 most significantly correlated genes with ZC3H12A.

Rank #	Gene	r	*P* value	Rank #	Gene	r	*P* value
1	NFKBIZ	0.6792	2.98E-31	26	COL9A2	0.4253	4.04E-11
2	CXCL2	0.6606	4.42E-29	27	XDH	0.4221	5.85E-11
3	CXCL3	0.6577	9.11E-29	28	SBNO2	0.4135	1.55E-10
4	CXCL1	0.6521	3.80E-28	29	DUOX2	0.4131	1.62E-10
5	SAA2	0.6043	2.19E-23	30	COMP	-0.4122	1.78E-10
6	SAA1	0.5995	5.95E-23	31	TNFAIP3	0.4053	3.82E-10
7	SAA4	0.5987	6.97E-23	32	MGP	-0.4039	4.44E-10
8	CCL20	0.5986	7.10E-23	33	TNIP3	0.4034	4.70E-10
9	SOCS1	0.5756	6.68E-21	34	CCL28	0.4019	5.49E-10
10	CEBPD	0.5549	3.01E-19	35	RTEL1	0.4009	6.08E-10
11	CFB	0.5503	6.74E-19	36	LRG1	0.4008	6.16E-10
12	LCN2	0.5285	2.69E-17	37	GRP	-0.3976	8.69E-10
13	NOS2A	0.5238	5.68E-17	38	PRKD1	-0.3897	1.98E-09
14	MAP3K8	0.5177	1.51E-16	39	ITGBL1	-0.3869	2.63E-09
15	BCL3	0.5140	2.67E-16	40	SLIT2	-0.3853	3.10E-09
16	EBF2	0.4772	5.72E-14	41	MUC1	0.3846	3.33E-09
17	SLC37A1	0.4647	3.07E-13	42	ST5	0.3825	4.11E-09
18	IL26	0.4587	6.77E-13	43	CCR6	0.3806	4.98E-09
19	PIM3	0.4478	2.71E-12	44	MAP2K6	0.3785	6.16E-09
20	VNN3	0.4432	4.76E-12	45	FCRL5	0.3745	9.11E-09
21	IKBKE	0.4410	6.20E-12	46	REG3A	0.3727	1.09E-08
22	VNN1	0.4341	1.43E-11	47	CASP1	0.3724	1.12E-08
23	IL17A	0.4320	1.84E-11	48	HTR2B	-0.3704	1.35E-08
24	DUOXA2	0.4298	2.40E-11	49	TRPC1	-0.3702	1.38E-08
25	CASP10	0.4269	3.36E-11	50	ZFP36	0.3684	1.65E-08

Supplementary Table 5. Gene expression correlation of *ZC3H12A* with immune checkpoint molecules.

Immune checkpoints	r	P value
PD-1 (PDCD1)	0.2225	8.68E-04
PD-L1 (CD274)	0.2091	1.78E-03

Supplementary Table 6. Gene expression correlation of ZC3H12A with either pro-apoptotic or anti-apoptotic genes*.

Pro-apoptotic gene	r	*P value*	Anti-apoptotic gene	r	*P value*
BAD	-0.0680	0.315	BCL2A1	-0.0397	0.558
DEDD2	0.1073	0.112	BCL2L1	-0.1217	0.071
FAS	0.0337	0.618	BCL3	0.5140	2.67E-16
RIPK2	-0.1002	0.138	BIRC3	0.2867	1.50E-05
			RELB	0.0232	0.732

* the apoptotic genes were obtained from the reference (Lu et al., Cancer Res, 2016)

SUPPLEMENTARY MATERIALS

Supplementary Table 7. Gene expression correlation of ZC3H12A with proinflammatory or angiogenic genes*.

Proinflamatory gene	r	*P* value	Angiogenic gene	r	*P* value
IL6	0.1154	0.087	VEGFA	0.1821	6.62E-03
IL8	0.1124	0.096	CD31	-0.0988	0.124
IL1B	0.3055	3.71E-06			
IL2	-0.0189	0.780			
IL12B	0.0695	0.304			

* these genes were obtained from the references (Marona et al., Cancer Res, 2017)

Supplementary Table 8. Predictive characteristics for ZC3H12A as stage I biomarker in colorectal cancer from the TCGA, GSE14333, and Shanghai patient cohorts.*

Cohort	Platform	Sensitivity, %	Specificity, %	Positive-predictive value, %	Negative-predictive value,%
TCGA	RNA-seq	63.0	71.4	36.7	88.0
GSE14333	Microarray	61.4	78.4	33.8	91.9
Shanghai	rt-PCR	85.7	80.6	23.1	98.8
	IHC	85.7	66.0	14.6	98.6

*The expression cutoff was derived by receiver operating characteristic curves with Youden's index.

SUPPLEMENTARY MATERIALS

Supplementary Figure 1

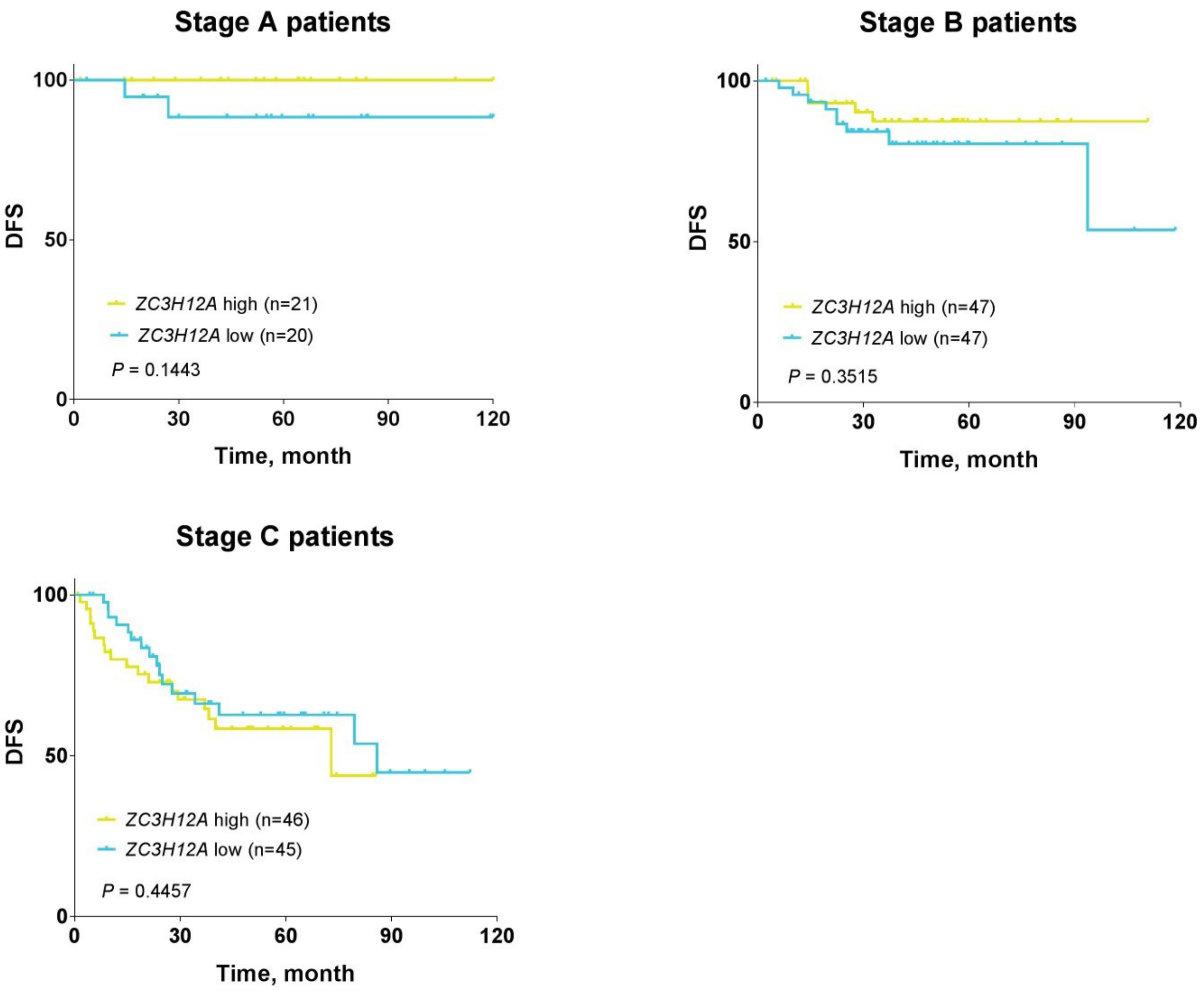

Supplementary Figure 1. Correlation of ZC3H12A mRNA expression with disease-free survival within stage A, or B, or C patients, separately, in the validation cohort (GSE14333). Patients were stratified into either high or low group based on ZC3H12A median expression in each category. The DFS data were not available for the stage D patients.

SUPPLEMENTARY MATERIALS

Supplementary Figure 2

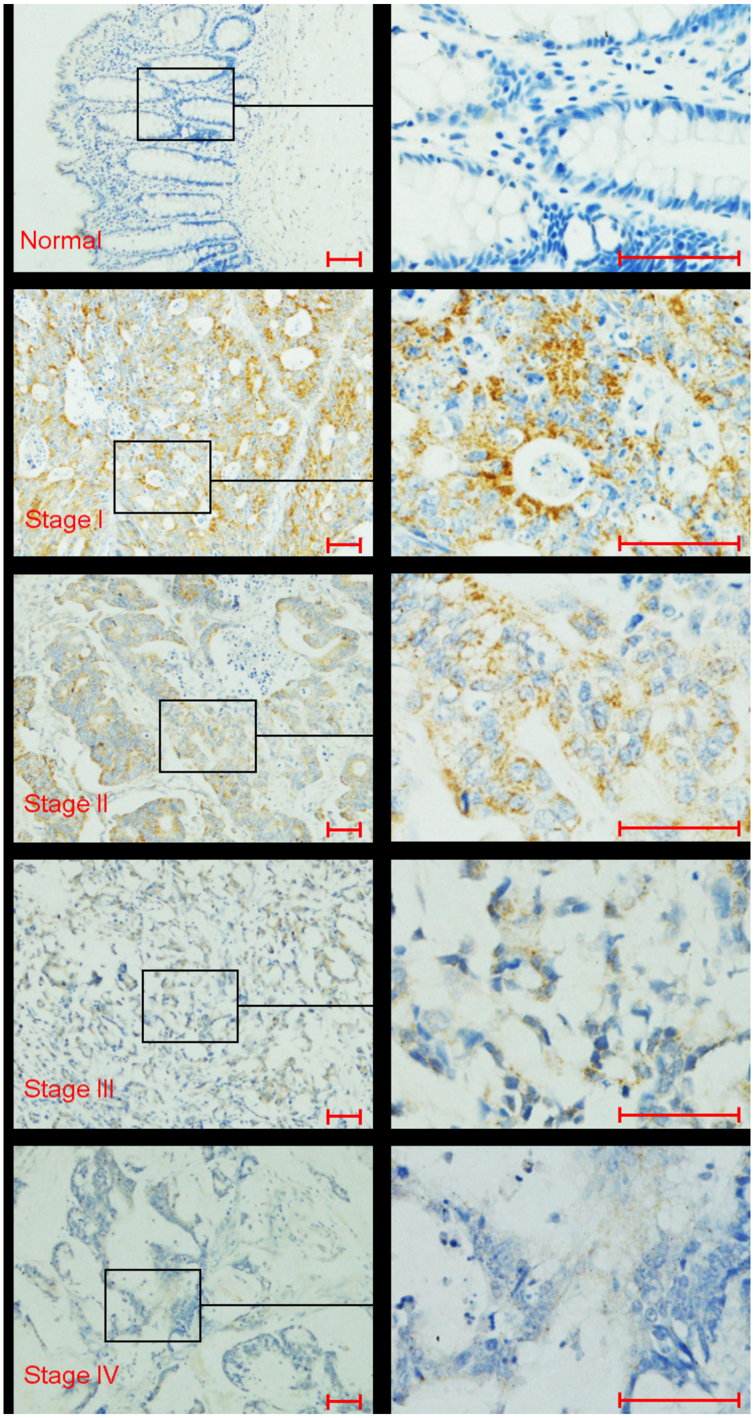

Supplementary Figure 2. An enlarger version of the panel Fig. 4B is shown for the purpose of clarity.

SUPPLEMENTARY MATERIALS

Supplementary Figure 3

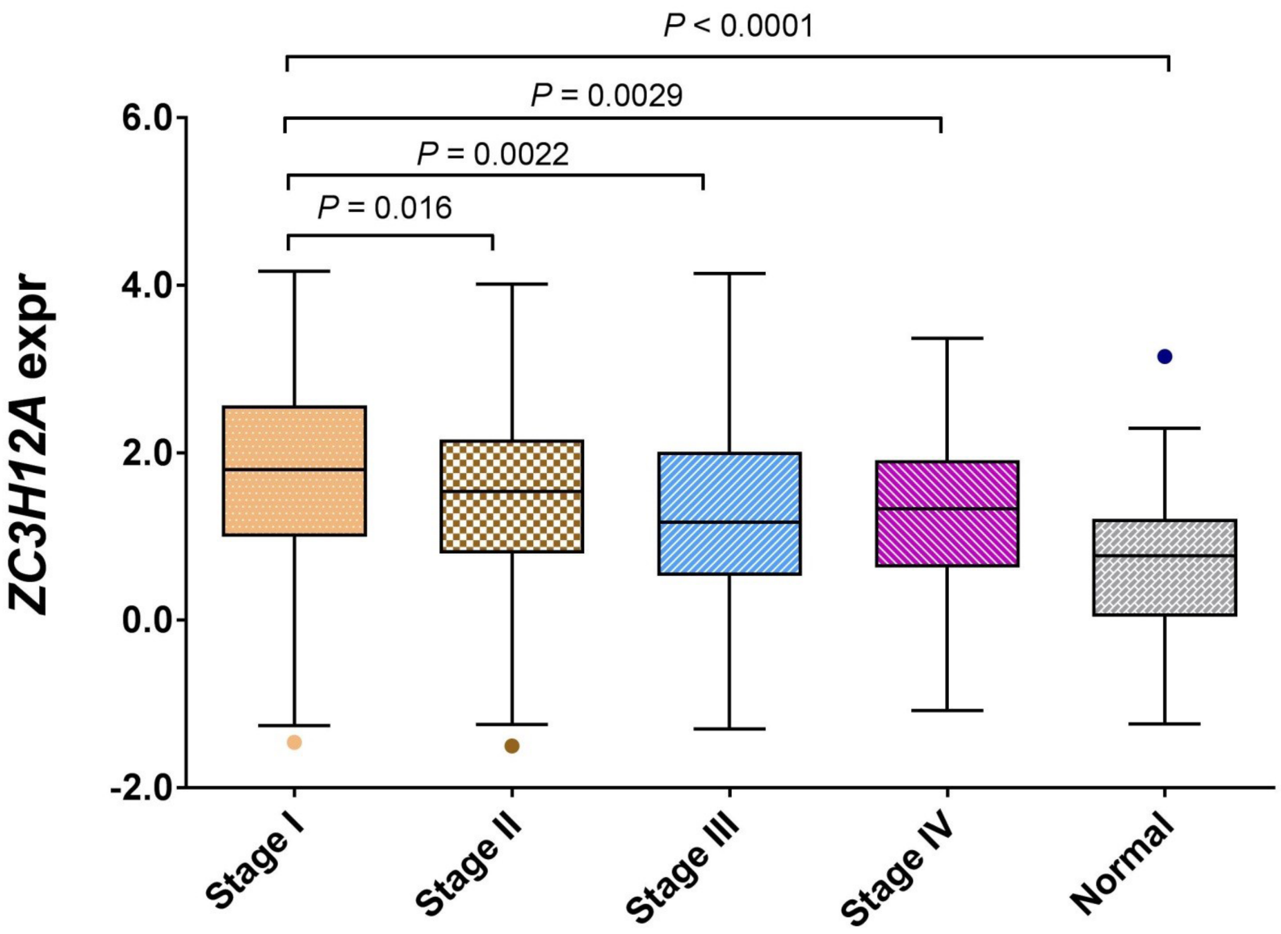

Supplementary Figure 3. Comparison of ZC3H12A mRNA expression among normal samples and CRC tumor samples at different stages. Data were presented in the bar-and-whiskers plots (center line, median; box limits, upper and lower quartiles; whiskers 1.5 x interquartile range; and points, outliers).
